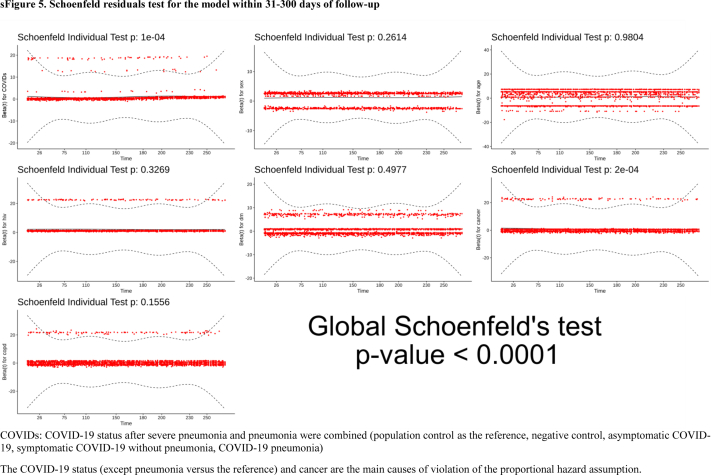# COVID-19 pneumonia and the subsequent risk of getting active pulmonary tuberculosis: a population-based dynamic cohort study using national insurance claims databases

**DOI:** 10.1016/j.eclinm.2023.101825

**Published:** 2023-01-20

**Authors:** Ponlagrit Kumwichar, Virasakdi Chongsuvivatwong

**Affiliations:** Department of Epidemiology, Faculty of Medicine, Prince of Songkla University, Kanjanavanich Rd, Kho Hong, Hat Yai District, Songkhla, 90110, Thailand

**Keywords:** COVID-19, Tuberculosis, Risk

## Abstract

**Background:**

A three-fold increase in the incidence of detecting pulmonary tuberculosis (PTB) in patients hospitalised with COVID-19 pneumonia compared with that in the general population was recently reported; however, this finding may be due to admission bias in the diagnostic investigation. The current cohort study aimed to estimate the risk of having detectable active PTB after SARS-CoV-2 infection.

**Methods:**

Insurance claims data in lower Southern Thailand from the 12th regional National Health Security Office, Thailand, were used. Inpatient and outpatient electronic medical records were linked using encrypted identification numbers. Records of individuals aged ≥18 years from 1 April to 30 September 2021 were retrieved to form a dynamic cohort. Exposure status was based on SARS-CoV-2 investigation and pneumonia status: population control (general population who had never been tested), negative reverse transcription-polymerase chain reaction (RT-PCR) control, asymptomatic COVID-19, symptomatic COVID-19 without pneumonia, and COVID-19 pneumonia groups. They were tracked in the databases for subsequent bacteriologically confirmed PTB until 31 March 2022.

**Findings:**

Overall, 4,241,201 individuals were recruited in the dynamic cohort and contributed 3,108,224, 227,918, 34,251, 10,325, and 14,160 person-years in the above exposure groups, respectively. Time-varying Cox's regression was conducted using population control as reference. Hazard ratios (95% CIs) of the negative control, asymptomatic, symptomatic COVID-19 without pneumonia, and pneumonia groups were 1.58 (1.08, 2.32), 1.00 (0.25, 4.01), 2.98 (0.74, 11.98), 9.87 (5.64, 17.30) in the first 30 days and 0.97 (0.81, 1.15), 1.41 (0.92, 2.17), 3.85 (2.42, 6.13), and 7.15 (5.54, 9.22) thereafter, respectively.

**Interpretation:**

Having had COVID-19 pneumonia, as opposed to the general population status, was strongly associated with a higher hazard of detectable active PTB. In tuberculosis endemic areas, patients with COVID-19 pneumonia should be closely followed up to reduce PTB-related burden.

**Funding:**

The 10.13039/100000061Fogarty International Center and the 10.13039/100000060National Institute of Allergy and Infectious Diseases of the 10.13039/100000002National Institutes of Health supported the article processing charges under Award Number D43TW009522.

**Research in context**

**Evidence before this study**

Tuberculosis (TB) infection (TBI) can progress to active TB disease after an immunosuppressive event. Recently, multiple authors have reported that patients with COVID-19 have decreased levels of functional T-cells during the illness period. In practice, this depletion of immunity might lead to an increased incidence of active TB in populations with a high TB prevalence. We searched for studies exploring the association between COVID-19 and tuberculosis in PubMed using the terms "Tuberculosis"[Mesh] AND ("COVID-19"[Mesh]), without any filter or limitation, on 30 June 2022. No cohort study has addressed the risk of having active TB in patients with COVID-19 using an adequate sample size and a sufficient follow-up period.

**Added value of this study**

In this cohort study, we analysed time-to-event outcomes from SARS-CoV-2 infection to detectable active pulmonary TB (PTB) and compared it with the concurrent untested population in national insurance claims databases. The exposure groups were those with negative reverse transcription-polymerase chain reaction (RT-PCR), asymptomatic COVID-19, symptomatic COVID-19 without pneumonia, and COVID-19 pneumonia, while the reference group was the general population who had never been tested. Because patients with COVID-19 who are hospitalised are more likely to be diagnosed with PTB during hospitalisation, focusing on co-infections without properly accounting for time may result in a spurious association. To overcome this bias, we performed a time-varying analysis by separating the relationship into two phases: 0–30 days of COVID-19 diagnosis and 31–300 days of that. The hazard ratio (95% CI) of COVID-19 pneumonia was, as expected, very high (9.87 [5.64, 17.30]) in the first phase. The hazard ratio remained high in the second phase (7.15 [5.54, 9.22]).

**Implications of all the available evidence**

The association between COVID-19 pneumonia and the hazard of detectable active PTB indicates a need for intensive TB surveillance for patients with COVID-19 pneumonia in TB-endemic areas. The frequency of follow-up for PTB and the requirement of providing tuberculosis preventive treatment among individuals with prior COVID-19 pneumonia need to be clarified.

## Introduction

Tuberculosis (TB), a critical global infectious disease (GID), has been superimposed by the COVID-19 pandemic since 2020.^1^ In low- and middle-income countries (LMICs), information about the risk of getting active pulmonary tuberculosis (PTB) would guide health programmes on active surveillance of TB among post-COVID-19 patients.^2^ However, no study has examined the incidence of TB at the population level.^3^ Partly, this has been limited by the quality of health service databases in LMICs where TB is endemic.^4^

Thailand, a middle-income country, has achieved universal health coverage since 2002.^5^ National claims databases run by the National Health Security Office (NHSO) have been developed to perform transactions and audits of payment since the programmes' inception. Under the Thai Universal Health Coverage (UHC), Social Security Scheme, and Civil Servant Medical Benefit Scheme,^6^ all investigation and treatment costs of patients with communicable diseases, including TB and COVID-19, are covered.^7^^,^^8^ The data are delivered electronically from both private and public hospitals and for both in- and outpatients. Thus, the national claims databases of the NSHO are valuable for examining the association between these two GIDs.

The spectrum of PTB starts from tuberculosis infection (TBI), which is not contagious (dormant bacilli),^9^ to subclinical PTB with undetectable or detectable bacilli, and ends in active PTB with signs and symptoms, in which the bacilli are detected.^10^ In Thailand, subclinical PTB is often coincidentally detected during investigations for other purposes, and clinically active PTB patients visit healthcare centres because of signs and symptoms.^11^

COVID-19 pneumonia usually causes excessive cellular immune responses, resulting in functional exhaustion and T-cell depletion,^12^, ^13^, ^14^, ^15^ which can increase the risks of progression from TBI to active PTB.^12^, ^13^, ^14^, ^15^, ^16^ In contrast, individuals with asymptomatic or mildly symptomatic COVID-19 tend to have an adequate adaptive immune response, and they resume balanced cellular immune activity.^17^, ^18^, ^19^, ^20^ A hospital-based study in South Africa reported 1.5% COVID-19/PTB co-infection among patients with COVID-19 pneumonia^21^ representing a 3-fold increase in this incidence of pulmonary tuberculosis (PTB) compared with that in the general population.^22^ However, it gave no clear information on the sequence of PTB and SARS-CoV-2 infection. Without a cohort study, the findings may be attributed to a diagnostic bias (Berkson's bias).^23^ Patients with subclinical PTB or active PTB have a higher chance of being detected if they have a positive test for SARS-CoV-2 infection and are hospitalised. To avoid Berkson's bias, there is a need to conduct a cohort study to obtain an explicit sequence of these two infectious diseases with a reasonable period in between.

The objective of this study was to compare the incidence of detectable active PTB in three groups of COVID-19 patients, namely COVID-19 pneumonia (our main group of interest), symptomatic COVID-19 without pneumonia, and asymptomatic COVID-19, against that in the general population who have never undergone SARS-CoV-2 testing (population control). This study aimed to partially filled the knowledge gap regarding the progression from TBI to active PTB after SARS-CoV-2 infection. The study findings would have implications for TB surveillance among LMICs where TB is still endemic.

## Methods

### Study design

This is a retrospective cohort study based on the aforementioned claims databases where all groups of patients with COVID-19 were registered. The outcome was whether and how soon these patients would be detected as having active PTB after diagnosis of SARS-CoV-2 infection.

#### Setting

**Fig. 1:**
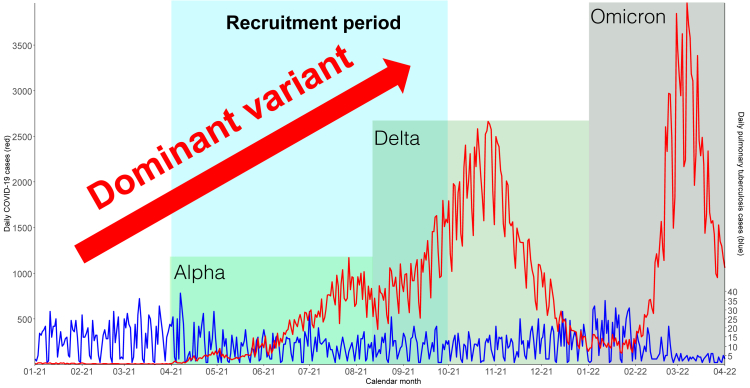
Incidence of COVID-19 and pulmonary tuberculosis in the study setting generated by data from the 12th regional National Health Security Office.

We defined the COVID-19 pandemic period between 1 April and 30 September 2021 (shaded area in Fig. 1) as the recruitment period. Individuals with respiratory symptoms or under contact tracing^24^ must undergo a reverse transcription-polymerase chain reaction (RT-PCR) test to confirm the SARS-CoV-2 infection.^25^ All individuals undergoing RT-PCR were assessed for high risk of severe COVID-19 (listed in Appendix Table S1).^26^ However, after 30 September 2021, Antigen Test Kits (ATKs) were introduced in the COVID-19 control programme to cover the overwhelming number the tests required.^27^ Thereafter, we ended the recruitment.

Fig. 1 shows the COVID-19 situation in the study area, which was dominated sequentially by the Alpha, Delta, and Omicron variants.^28^ During our recruitment period (shaded area in Fig. 1), the incidence of COVID-19 was rising due to the Alpha variant and the emergence of the Delta variant. The daily average COVID-19 incidence was 522 cases. Until 1 December 2021, there was no reinfection of SARS-CoV-2. For PTB, the notified number of bacteriologically confirmed PTB was approximately half that of the pre-COVID-19 period, with a relatively uniform daily average incidence of 10 cases. After the surge in the incidence of SARS-CoV-2 infection in April 2022, all TB services were disrupted. We, therefore, terminated the study on 31 March 2022. The outcome was whether the person recruited had detectable active PTB until the study was terminated.

**Figure S1:**
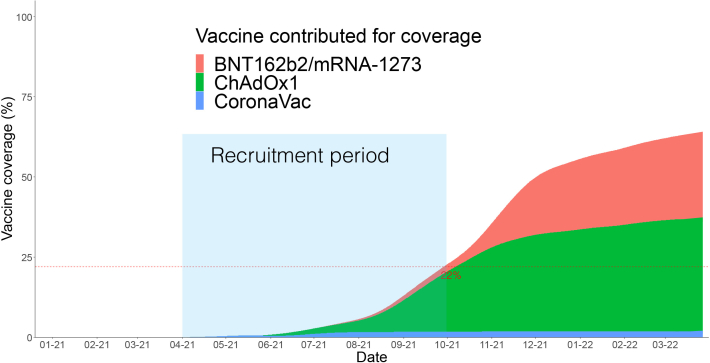
Vaccine coverage over time.

Regarding COVID-19 vaccinations, the coverage of at least two doses of started from zero at the beginning of the recruitment period (1 April 2021) and reached 22% of the population at the end of that period (30 September 2021), as shown in Figure S1 in the Appendix. Priority groups for vaccination were health care personnel and individuals with any vulnerable underlying conditions (see Appendix Table S1).^29^

### Data source

According to the National Civil Registration,^30^ there were 4,991,211 citizens in the study area on 1 April 2021. We used the claims databases of the 12th regional NHSO, which records all the health service data of those people. All health service units received COVID-19 and TB care reimbursements from the NHSO. This enabled NHSO to obtain the health service data of all individuals in Thailand. The coverage of these two diseases in the database was audited and allowed no missing records.

During the recruitment period, all RT-PCR-positive patients were hospitalised at a regular or field hospital for at least 14 days to minimise transmission. Therefore, the severity of the cases was fully assessed from the hospital records, which were compiled into the NHSO databases. All the hospitalised patients underwent chest radiography, and oxygen saturation at room air was monitored for early detection of COVID-19 pneumonia.^26^ For PTB, all such cases were confirmed by bacteriologic evidence for TB either by Ziehl-Neelsen staining of a sputum sample or a cartridge-based nucleic acid amplification test (Xpert MTB/Rif, Cepheid, Sunnyvale, California, USA) and registered in the NHSO databases using ICD-10 code as A15.0–A15.9.

### Participants and data management for exposure status

This was a dynamic cohort in which individuals aged at least 18 years on 1 April 2021 were recruited. Inpatient (IP) and outpatient (OP) electronic medical records of all individuals were linked using encrypted identification numbers. They were then de-identified for personal data protection. We identified individuals who had a history of TB infection from their prescription record of isoniazid, rifampicin, or rifapentine. They and subjects with a positive RT-PCR result for SARS-CoV-2 infection before 1 April 2021 were excluded.

The individuals’ exposure status could change based on the RT-PCR test over time. Hence, the population could contribute their time at risk in the reference group (population control group for people who had never been tested) from 1 April 2021. Further, the individuals could participate in the negative control group from the first day of the investigation with a negative result of RT-PCR until they tested positive. They could be either asymptomatic COVID-19, symptomatic COVID-19 without pneumonia, COVID-19 pneumonia, or severe COVID-19 pneumonia, depending on their clinical course in the hospital.

Using e-claim codes, ICD-9-CM, ICD-10, OP, and IP data were mined to classify the COVID-19 status as person-time units for each exposure group. Initially, the individuals' person-time units were categorised into the reference, negative control (RT-PCR claimed without a positive test result), and positive RT-PCR groups. The individuals whose person-times contributed to the positive group were sub-classified based on the codes listed in Table S2. Noteworthily, COVID-19 pneumonia with superimposed bacterial pneumonia (ICD-10 code J13–J17) was also categorised into the COVID-19 pneumonia groups.

### Outcome

The outcome was a diagnosis of bacteriologically confirmed PTB (ICD-10 code A15.0–A15.9) in both the IP and OP databases after the positive RT-PCR result for SARS-CoV-2 infection. Although all Thai adult citizens were eligible to undergo free chest radiographs annually, they were not performed on all patients during the pandemic period. Thus, active TB assessment in our study needed to rely on the diagnosis of TB among OP and IP claims databases.

### Statistical analysis

A time-to-event analysis was employed in this dynamic cohort. Individuals had one or more inception points, as aforementioned. All the subjects were terminated from the analysis on their date of death or date of PTB diagnosis (the first record of A15). Other censorships included the change in the COVID-19 status (exposure group), SARS-CoV-2 reinfection confirmed by RT-PCR or ATK, and status after 300 days of follow-up. The anticipated follow-up time ranged approximately from 180 for the last recruited ones to 300 days for the earliest recruited ones. Data were visualised using the Nelson–Aalen method^31^ for the cumulative incidence of PTB to explore Berkson's bias in the early follow-up period.

As the possible progression of PTB by SARS-CoV-2 needs some time, we used a time-varying approach^32^ to check whether the hazard ratio of COVID-19 is higher in the early follow-up period or in a later period. The cut-point for time stratification was arbitrarily set at the 95th percentile of the length of hospital stay among patients with severe pneumonia. This later turned out to be 30 days. Beyond this cut-point, the likelihood of diagnosis bias (due to severe cases being over-investigated) would be substantially reduced.

In the model, to avoid over-complexity, we had the severity of SARS-CoV-2 time-varying, and all other potential non-time-varying confounders: age, sex, human immunodeficiency virus infection (HIV), diabetes mellitus (DM), any cancers, and chronic obstructive pulmonary disease (COPD)^11^^,^^33^, ^34^, ^35^, ^36^, ^37^, ^38^, ^39^, ^40^, ^41^ (these four underlying diseases are available in the database).

The equation for our time-varying Cox's regression is as follows:

$$\lambda \left(t|x\right)=\lambda_{0}\;\exp\left(\beta_{1}x_{n}+\beta_{2}x_{t}+\beta_{3}x_{t}\times \left(t>C\right)\right)$$

Where x are all explanatory variables comprising: $x_{t}$ (time-varying covariables), and $x_{n}$ (non-time-varying covariables). $\lambda_{0}$ is a hazard function of the referent group (when all covariables are coded as 0). $\beta_{1}$ are coefficients of $x_{n}$*. C* is the time cut-point (30 days). (t>C) is coded as 1 if the time was after 30 days and 0 otherwise. $\beta_{2}$ are sole coefficients of $x_{t}$ when *t* *≤* *C*. $\beta_{3}$ are added to $\beta_{2}$ to obtain a new set of coefficients for $x_{t}$ when *t > C*.

In practice, the software will directly compute the values of $\beta_{2}$ and ($\beta_{2}$ + $\beta_{3}$) and exponentiate them to get hazard ratios for $x_{t}$ for each period. Thus, we had coefficients (or hazard ratios) of the severity of SARS-CoV-2 infection for the first 30 days different from those in the following period. On the other hand, all the other confounders would have constant coefficients over time.

Since the association observed in the analysis might be explained by unmeasured confounders, we further computed “E-value” as the minimum strength of association between both COVID-19 pneumonia and detectable active PTB for an unmeasured confounder that can explain away the association between COVID-19 pneumonia and hazard of detectable active PTB.^42^ We used the formula for the hazard ratio with a rare outcome (prevalence <15%),^43^ which is considered to be the risk of getting PTB among the population. If the E-value obtained was implausibly high, it would be unlikely that an unmeasured confounder could explain away the association found in our analysis.

All the analyses were performed using the epiDisplay (version 3.5.0.2),^44^ survminer (version 0.4.9),^45^ tidyverse (version 1.3.1),^46^ and E-value (version 4.1.3)^47^ packages on R language and environment version 4.1.1 (R Core Team (2021). R: A language and environment for statistical computing. R Foundation for Statistical Computing, Vienna, Austria). A p-value of <0.05 was considered statistically significant.

### Ethics statement

Patient data was encrypted and de-identified for personalised anonymisation according to the Thai Personal Data Protection Act 2019, Thailand (PDPA). Data were received from the 12th Regional NHSO under a project approval granted by the Human Research Ethics Committee, Prince of Songkla University (REC 65-231-18-4). Informed consent was not required because the data obtained could not identify any individual.

### Role of the funding sources

The 10.13039/100000061Fogarty International Center and the 10.13039/100000060National Institute of Allergy and Infectious Diseases of the 10.13039/100000002National Institutes of Health supported the article processing charges under Award Number D43TW009522. The content is solely the responsibility of the authors and does not necessarily represent the official views of the National Institutes of Health.

## Results

### Population demographics

**Fig. 2:**
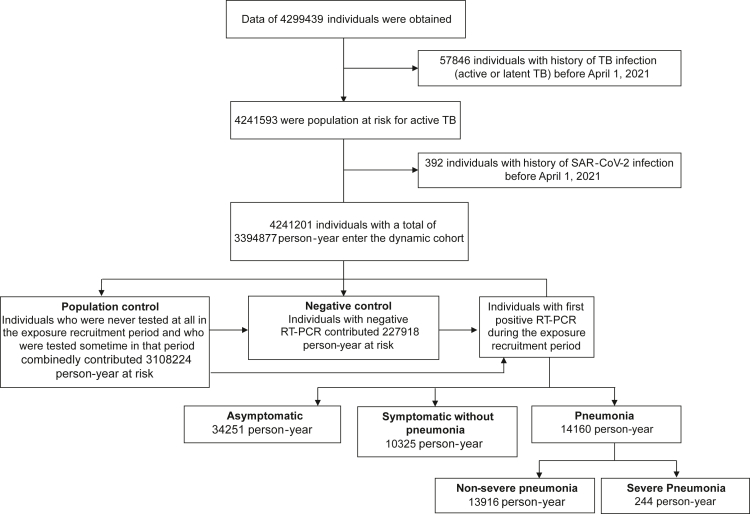
Study design and participants recruited.

The data of 4,299,439 individuals aged ≥18 years were retrieved. Fig. 2 illustrates that we excluded individuals with a history of TB infection or SARS-CoV-2 infection before 1 April 2021, resulting in 4,241,201 individuals in the population recruited into the dynamic cohort. The population control group contributed 3,108,224 person-years at risk. Individuals in the negative control group contributed 227,918 person-years at risk. Among patients with positive RT-PCR tests for the SARS-CoV-2 virus, person-years contributed by patients with asymptomatic COVID-19, symptomatic COVID-19 without pneumonia, and COVID-19 pneumonia were 34,251, 10,325, and 14,160, respectively. Severe pneumonia contributed 244 person-years among the patients with COVID-19 pneumonia. Among those individuals with severe pneumonia, 50.2% survived, and only six patients had subsequent PTB. This number was too small to report as a separate result; therefore, we considered and computed according to only one pneumonia group at the end.

Table 1 shows the distribution of the number of subjects in each COVID-19 status by other demographic and comorbidity variables. The majority were female. Older adults (>60 years old) accounted for one-fifth of the population but with a higher proportion of those in the COVID-19 pneumonia group. The same pattern of distribution was seen for both DM and COPD. As expected, new PTB was primarily detected in the control population. Overall, the median time interval between SARS-CoV-2 infection and detectable active PTB was 175 days (IQR between 97 and 237 days).

**Table 1:** Number of individuals and the percentage in different exposure groups.

	Population control (n = 3,818,612)	Negative control (n = 384,423)	Asymptomatic pneumonia (n = 54,286)	Symptomatic COVID-19 without pneumonia (n = 16,935)	COVID-19 pneumonia (n = 23,799)	Severe COVID-19 pneumonia (n = 818)
Demographics, n (%)						
Sex						
Male	1,766,347 (46.3)	172,101 (44.8)	15,734 (29.0)	4507 (26.6)	7526 (31.6)	335 (41.0)
Female	2,052,265 (53.7)	212,322 (55.2)	38,552 (71.0)	12,428 (73.4)	16,273 (68.4)	483 (59.0)
Age group						
18–40 years	1,745,880 (45.7)	201,771 (52.5)	41,316 (76.1)	11,833 (69.9)	10,773 (45.3)	169 (20.7)
41–60 years	1,285,773 (33.7)	128,483 (33.4)	10,247 (18.9)	3435 (20.3)	7380 (31.0)	283 (34.6)
>60 years	786,959 (20.6)	54,169 (14.1)	2723 (5)	1667 (9.8)	5646 (23.7)	366 (44.7)
Comorbidities						
HIV^a^	21,733 (0.6)	2261 (0.6)	206 (0.4)	58 (0.3)	115 (0.5)	3 (0.4)
Diabetes mellitus	245,024 (6.4)	25,981 (6.8)	1622 (3)	893 (5.3)	3394 (14.3)	247 (30.2)
Any cancer	70,780 (1.9)	7370 (1.9)	234 (0.4)	107 (0.6)	307 (1.3)	13 (1.6)
COPD^b^	45,615 (1.2)	5007 (1.3)	204 (0.4)	236 (1.4)	545 (2.3)	31 (3.8)

a HIV: human immunodeficiency virus infection.

b COPD: chronic obstructive pulmonary disease, COVID-19: Coronavirus disease 2019.

### Hazard of detectable active pulmonary tuberculosis

**Fig. 3:**
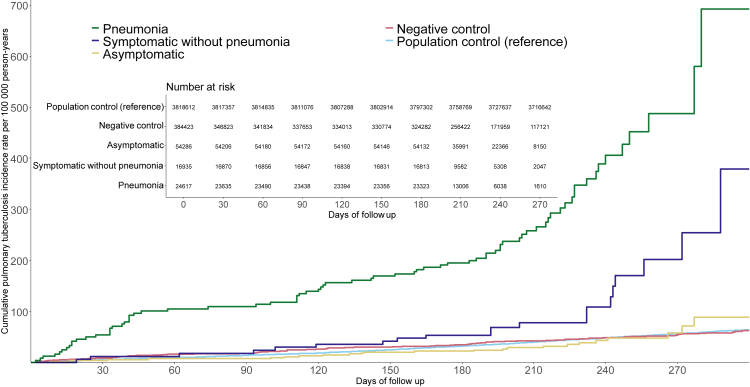
Pulmonary tuberculosis incidence over time stratified by five groups of exposure.

Fig. 3 illustrates the cumulative PTB incidence in person-years among individuals with different COVID-19 statuses. The cumulative incidence plot shows different shapes for subgroup severity. The reference, negative control, and asymptomatic groups had a relatively constant incidence of PTB over time, as their curve showed little variability over the study period. Obviously, the pneumonia group has a steeper slope at the beginning than in the follow-up period.

We used time-varying Cox's regression to handle the change in hazard ratios. As most of the hospitalised patients with COVID-19 were discharged after the 30th day of hospital stay (the 95th percentile of the length of hospital stay of patients with severe pneumonia), we finally stratified the person-time units into 30 days or less, and the remaining follow-up period. Diagnostics for the Cox model are shown in sFigure 2–5. The cumulative incidence curves of the COVID-19 pneumonia groups and the population control group are somewhat parallel, indicating a relatively constant hazard ratio (sFigure 3). As expected, p-values in some of the tests on an assumption of proportional hazard were significant in the late phase of the follow-up period due to the large sample size.

COVID-19 pneumonia was extraordinarily highly associated with the hazard of detectable active PTB with a hazard ratio (95% CI) of 9.87 (5.64, 17.30; Table 2) in the first 30 days. The hazard ratio (HR) was reduced to 7.15 (5.54, 9.22; Table 2) after the initial 30-day period. Similarly, there was a significant association between symptomatic COVID-19 without pneumonia and the hazard of detectable active PTB in the second period, but to a lower extent. The negative control group had a slightly increased hazard, but the HR was reduced thereafter, subsequently reaching the same hazard as that of the population control group. The patients with asymptomatic COVID-19 had no additional hazard of getting PTB.

**Table 2:** Pulmonary tuberculosis incidence in subgroups and adjusted hazard ratios of the severity of COVID-19 and the potential confounders.

	Subsequent tuberculosis cases per person-years	Tuberculosis incidence per 100,000 person-years (95% CI)	aHR (95% CI)	p-value
**Time-varying effect (COVID-19 status)**				
*0–30 days of follow up*				
Not under investigation (population control)	208/313,813	66 (57, 75)	1 (ref)	..
No infection (negative control)	30/29,026	103 (72, 147)	1.58 (1.08, 2.32)	0.019
Asymptomatic COVID-19	2/4458	44 (11, 175)	1.00 (0.25, 4.01)	0.995
Symptomatic COVID-19 without pneumonia	2/1389	144 (36, 575)	2.98 (0.74, 11.98)	0.125
COVID-19 pneumonia	13/1973	658 (382, 1131)	9.87 (5.64, 17.30)	<0.0001
*31–300 days of follow up*				
Not under investigation (population control)	2215/2,794,411	79 (75, 82)	1 (ref)	..
No infection (negative control)	140/198,892	70 (59, 82)	0.97 (0.81, 1.15)	0.696
Asymptomatic COVID-19	21/29,793	70 (45, 107)	1.41 (0.92, 2.17)	0.118
Symptomatic COVID-19 without pneumonia	18/8936	201 (126, 318)	3.85 (2.42, 6.13)	<0.0001
Pneumonia COVID-19	62/12,187	508 (396, 651)	7.15 (5.54, 9.22)	<0.0001
**Non-time-varying effects**				
*Sex*				
Female	749/1,836,777	40 (37, 42)	1 (ref)	..
Male	1962/1,558,100	125 (119, 130)	3.16 (2.90, 3.43)	<0.0001
*Age (years)*				
18–40	865/1,580,209	54 (50, 57)	1 (ref)	..
41–60	1075/1,136,834	94 (88, 99)	1.46 (1.34, 1.61)	<0.0001
>60	771/677,835	113 (105, 121)	1.53 (1.37, 1.70)	<0.0001
*Underlying diseases*				
HIV				
No	2572/3,375,716	76 (73, 78)	1 (ref)	..
Yes	139/19,161	725 (614, 855)	8.44 (7.11, 10.03)	<0.0001
Diabetes mellitus				
No	2261/3,178,673	71 (68, 73)	1 (ref)	..
Yes	450/216,204	208 (189, 228)	2.50 (2.24, 2.78)	<0.0001
Any cancer				
No	2569/3,354,787	76 (73, 78)	1 (ref)	..
Yes	142/40,090	354 (300, 417)	2.05 (1.71, 2.46)	<0.0001
Chronic obstructive pulmonary disease				
No	2586/3,332,890	77 (74, 80)	1 (ref)	..
Yes	125/61,987	201 (168, 239)	2.54 (2.13, 3.03)	<0.0001

aHR: adjusted hazard ratio, CI: confidence interval, COVID-19: coronavirus disease 2019.

### Sensitivity analysis

Finally, we conducted sensitivity analysis to check whether the HR of 7.15 could possibly be explained away by an unmeasured confounder using E-value. The E-value (95% CI) from the software was 13.78 (10.56, NA). The lower limit of the confidence interval was far away from the null (E-value = 1). It sounds implausible to have an unmeasured confounder that would explain away the association between COVID-19 pneumonia and the hazard of detectable active PTB.

## Discussion

The cumulative incidence of PTB among patients with SARS-CoV-2 infection was steep at ten-fold of the population control in the early period (within 30 days after infection), and it declined to six-fold thereafter. In the second period, patients with symptomatic COVID-19 (both with pneumonia and without pneumonia) demonstrated an increased hazard of detectable active PTB. Also, the negative control had a slightly increased hazard in the early period. These would be patients who had PTB without SARS-CoV-2 co-infection. However, the degree of increase in the hazard subsided later in the follow-up. Only COVID-19 pneumonia was persistently associated with the hazard of detectable active PTB.

With accurate timing of the events of SARS-CoV-2 infection and the ascertainment of active PTB, our data removed the doubt on the chronological sequence found in the previous study.^21^ The differences in the hazard for detectable active PTB among groups with positive RT-PCR results for the SARS-CoV-2 virus may be explained by two main arguments: biological effects of SARS-CoV-2 infection and the difference in the chance of being investigated for PTB, called Berkson's bias.^23^ The correlation between TB susceptibility and SARS-CoV-2 infection could be potentially ruled out as the underlying diseases were already included in the multivariable model.

Several studies have demonstrated that T cells, especially CD8^+^, play a significant role in the defence against TB.^12^, ^13^, ^14^, ^15^, ^16^ Two studies in China asserted that there is a significantly decreased number of CD8^+^ cells at the time of SARS-CoV-2 infection.^48^^,^^49^ The depletion of CD4^+^ and CD8^+^ in the early period of the SARS-CoV-2 infection has a dose–response relationship with the severity of COVID-19.^48^^,^^49^ Thus, the severity of COVID-19 could be a surrogate variable for the T-cell count affecting clinical presentation. Additionally, a study in China reported that 73.6% of patients with pneumonia could recover their T-cell numbers within approximately 30 days, whereas the remaining 26.4% had prolonged T-cell depletion.^50^ Decreased cellular immune response due to T-cell depletion could mediate the increased risk of getting active TB. These findings could explain why patients with COVID-19 pneumonia may be at increased risk of subsequent PTB even if their COVID-19 symptoms are cured.

On the other hand, an in vitro study reported that patients with COVID-TB co-infection have a decreased response to SARS-CoV-2 and an intact *Mycobacterium tuberculosis* (Mtb) response. Moreover, COVID-19 patients with latent TB infection (LTBI) seem to have the ability to react to both SARS-CoV-2 and Mtb antigens.^51^ Thus, the increased hazard of detectable active PTB in COVID-19 pneumonia was less likely to be from reactivation of LTBI due to the COVID-LTBI interaction but more likely to be from the weakened immune system as the aforementioned. However, as we could not identify individuals' spectrum of TB infection before SARS-CoV-2 infection, this hypothesis could not be verified.

Regarding Berkson's bias, patients with COVID-19 pneumonia were diagnosed based on radiography findings. The increased access to radiography in COVID-19 cases could increase the chances of detecting PTB in these patients. In contrast, individuals in the population control and negative control groups were devoid of this chance unless they had a lower respiratory symptom or were scheduled for a routine chest radiography before any operation. This bias was likely to reduce after the patients were discharged. Thus, Berkson's bias could explain only the relatively severe cases in the initial 30-day period of follow-up.

Steroids are well known to impair cell-mediated immunity.^52^ During hospitalisation, the use of steroids in Thailand was limited only to patients with severe pneumonia. Almost half of them passed away before the follow-up time was sufficient to detect active PTB. Hence, steroid use was unlikely to be a reasonable explanation.

Whether the bacteriologically confirmed PTB cases were newly acquired or reactive cannot be established. While SARS-CoV-2 is different from TB, it probably weakens individuals with TB infection and accelerates their progression toward active PTB. Our study in the pre-COVID-19 period found that PTB was mostly misdiagnosed as unspecified pneumonia.^11^ During the COVID-19 pandemic, some patients with COVID-19 pneumonia might already be active PTB. However, such cases would be a minority as we advocated the health policy to reduce delayed diagnosis.^11^ In this study, patients with PTB were diagnosed after being discharged from the hospital in the median of 175 days. They were more likely to have active PTB during their post-COVID-19 period.

The study's strength is the completeness of COVID-19 and PTB diagnoses data based on a large population by the law for reimbursement that was followed up for at least 180 days. The major biological risk factors for PTB, i.e., age, sex, HIV infection, DM, cancers, and COPD, were already controlled for. Thus, they could not explain this independent association between COVID-19 and PTB.

For the limitation, we did not control for behaviour and other social variables such as time spent in a crowded area, economic status, and poor respiratory hygiene. While those data were unavailable, our sensitivity analysis revealed a very high E-value of 13.78, which means that the unmeasured confounder must have an explicitly high strength of association with both COVID-19 pneumonia and PTB (risk ratio >13.78) to explain away our findings. To date, no such confounder has been identified in published literatures. Berkson's bias has been reduced but not completely eliminated by time-varying Cox's regression. Although PTB was detected on chest radiographs after SARS-CoV-2 infection, it might have existed long before the pandemic. Temporal sequence of these two infections therefore could not be confirmed. The results must be interpreted with caution.

In conclusion, people who recently survived COVID-19 pneumonia in a TB endemic area should receive special attention regarding the robust association between COVID-19 pneumonia and the hazard of detectable active PTB. Further studies on this population on TB infection and the cost-utility for screening and TB preventive therapy are needed.

## Contributors

PK and VC initiated and designed the study. PK and VC directly accessed and verified the underlying data in this article. PK obtained, combined, and transformed data for analysis. PK and VC analysed the data, interpreted the results, and wrote the manuscript. Both authors were involved in the decision to submit and agreed to publish the paper.

## Data sharing statement

The data of the dynamic cohort and R codes used in the analysis are available in a GitHub repository. For the GitHub repository for this study see https://github.com/ponlagrit/covid_tb.

## Declaration of interests

We have no conflicts of interest to declare.

## Figures and Tables

**Figure S2:**
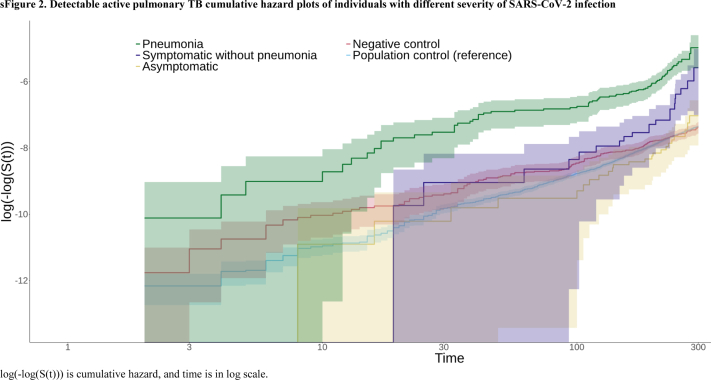


**Figure S3:**
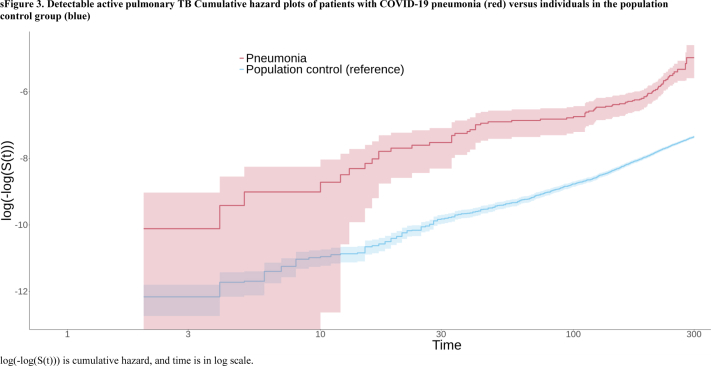


**Figure S4:**
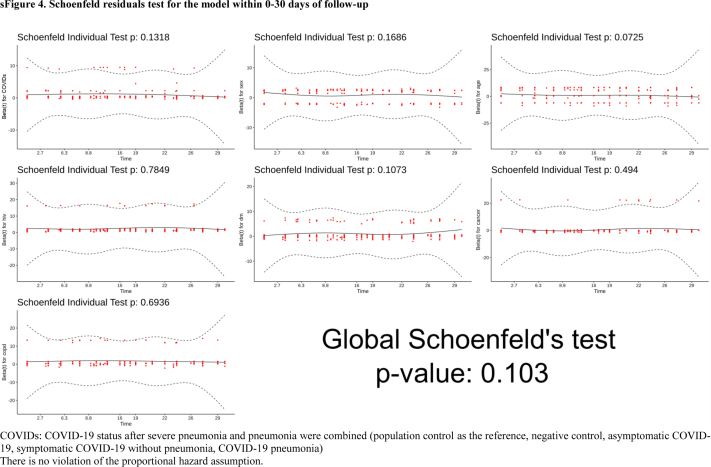


**Figure S5:**